# Validation of chemical inactivation methods for Select Agent compliance using HPAIV and low-pathogenicity influenza A virus in tissues and cultured cells

**DOI:** 10.1371/journal.pone.0362117

**Published:** 2026-11-06

**Authors:** Yuekun Lang, Anthony Griffiths

**Affiliations:** 1 Pathobiology and Integrative Biomedical Sciences, University of Missouri, Columbia, Missouri, United States of America; 2 Laboratory for Infectious Disease Research, University of Missouri, Columbia, Missouri, United States of America; 3 Department of Microbiology and Immunology and Bond Life Sciences Center, University of Missouri, Columbia, Missouri, United States of America; National Taiwan Ocean University, TAIWAN

## Abstract

The US Federal Select Agent program requires that inactivation procedures be validated before treated materials can be removed from biocontainment for downstream use. Although H5 avian influenza viruses are subject to temporary exemption from the Select Agent regulatory requirements, validated inactivation workflows remain critical for safe downstream handling of HPAIV-containing materials. Common treatments, including TRIzol, TRIzol LS, formalin, and ice-cold methanol, are frequently used to facilitate virus inactivation and downstream processing. However, facility-specific inactivation validation is required for Select Agent workflows and for safe handling of HPAIV-containing materials. In this study, HPAIV (H5N1)-infected samples were used to validate inactivation of material generated in cell culture, and low-pathogenicity (H1N1)-infected lung homogenates were used to validate inactivation of animal-derived tissues. Following inactivation, column-based buffer exchange was used to remove cytotoxic inactivants before viability testing. This step did not significantly reduce recovery of infectious virus, supporting the sensitivity of this approach. In inactivation tests, residual infectious virus was assessed by plaque assay and serial blind passages. No infectious virus was detected following any of the tested conditions. These data validate several procedures for inactivating influenza A virus-containing cell cultures, fluids, and tissue samples and provide a framework for facility-specific validation of similar workflows.

## Introduction

The Federal Select Agent Program oversees the possession, use and transfer of select agents and toxins, which pose a threat to public, animal, or plant health (7 C.F.R. Part 331, 9 C.F.R. Part 121, and 42 C.F.R. Part 73; http://www.selectagents.gov). The program requires that inactivation procedures be validated before treated Select Agents can be removed from biocontainment for use at a lower containment level [1]. In practice, this means that the procedures performed to render the Select Agent non-viable in a sample must be confirmed by each facility through a viability testing protocol [2].

Highly Pathogenic Avian Influenza (HPAI) virus is a highly infectious IAV that is often fatal to domestic poultry and, less commonly, people [3]. Some HPAIVs are regulated as USDA Select Agents. In response to the ongoing H5 avian influenza outbreak, USDA APHIS issued a temporary exemption on June 6, 2024, exempting H5 avian influenza viruses from the requirements for three years. This was intended to facilitate outbreak response and research while meeting shipping requirements. Despite the exemption, validated inactivation procedures are critical for laboratories handling HPAIV-containing materials, particularly when they are removed from biocontainment for downstream analyses.

Influenza virus belongs to the *Orthomyxoviridae* family, and particles are enveloped and contain a negative-sense, single-stranded RNA genome [4]. Many downstream analyses involving HPAIV require processing to inactivate infectious virus particles. For example, chemical reagents like 10% neutral buffered formalin (NBF) are routinely used for histological preservation, ice-cold methanol is used for cellular fixation, and TRIzol or TRIzol LS are employed for nucleic acid isolation [5–11]. Although these reagents are expected to inactivate enveloped viruses, Select Agent regulations require facility-specific validation under the conditions in which the reagents are used. Previous studies have evaluated the susceptibility of influenza A viruses, including HPAIV, to commonly used chemical inactivation and sample-preparation reagents, and prior work has also described approaches for verification of inactivation procedures in high-containment laboratories [8,12–14]. The present study extends this work by integrating multiple sample types (infected cultured cells, virus-containing fluids, and infected lung homogenates), removal of residual cytotoxic inactivants by column-based buffer exchange, and a sensitive infectivity endpoint incorporating serial blind passage. This design provides a practical framework for facility-specific validation of influenza A virus inactivation workflows.

In this study, we developed and evaluated viability testing protocols for chemical inactivation of IAV-containing infected cell cultures, virus-containing fluids, and infected mouse lung homogenates. Viral inactivation was assessed using plaque assays and serial blind passages in permissive MDCK cells. These experiments established validated procedures for the safe downstream handling of chemically inactivated IAV samples outside BSL-3 containment.

## Results

### Determination of limit of detection (LOD)

To define the sensitivity for the detection of H5N1 infectious particles following the various inactivation protocols, an LOD analysis was performed.

Cells infected with 1 and 10 plaque forming units (PFU) of H5N1 virus showed cytopathic effects (CPE) following the first blind passage (Fig 1, images highlighted in the red frame), while cells infected with 0.1 PFU showed obvious CPE following the second blind passage. Neither uninfected cells nor cells infected with 0.01 PFU of H5N1 virus showed CPE through the end of the experiment.

**Fig 1:**
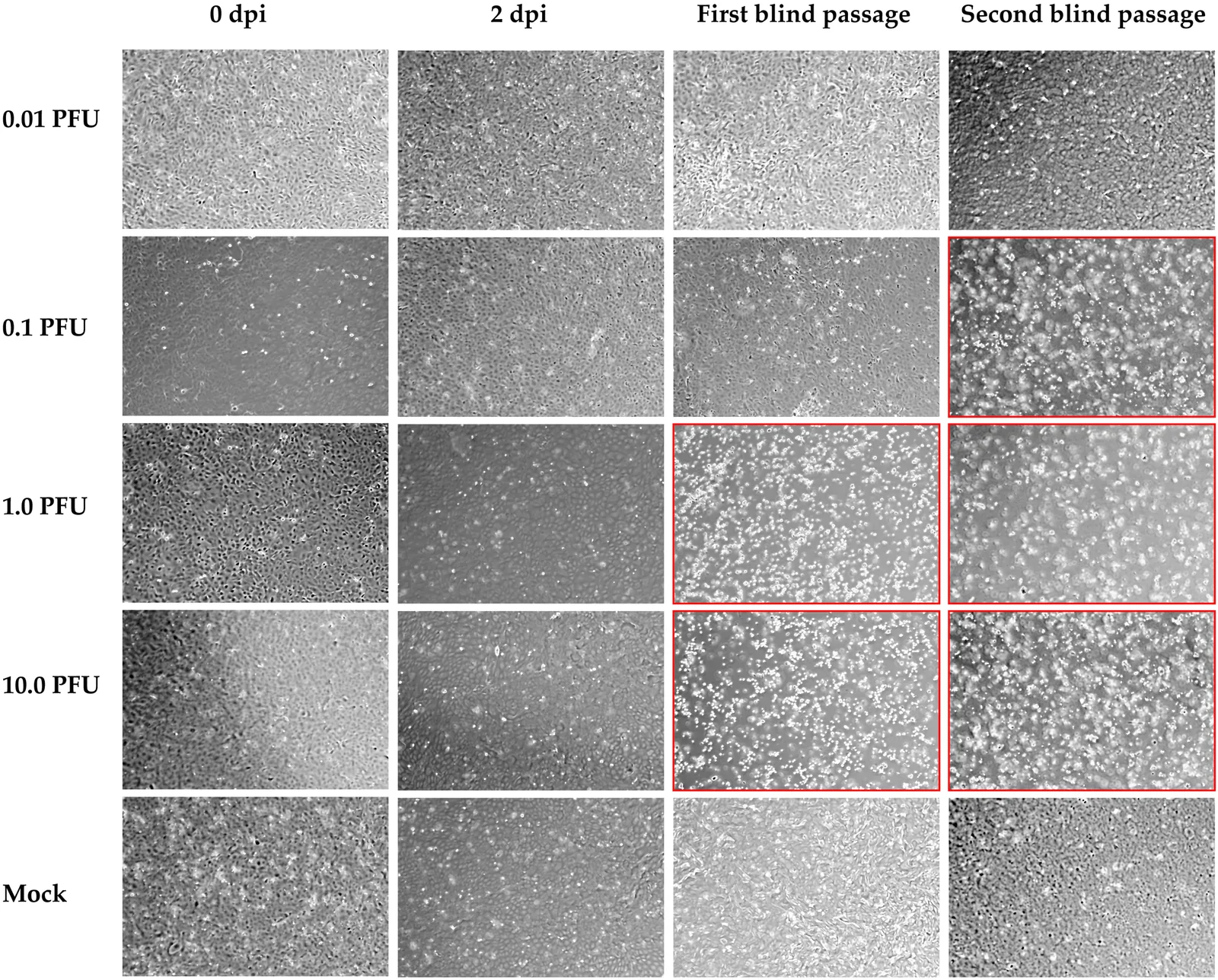
H5N1 limit detection on MDCK cells without pretreatment. Representative microscope images (10x) of infected cells with different doses of H5N1 at 2 days post-infection (dpi), following the first blind passage, and following the second blind passage. Images with CPE are outlined in red. CPE was evaluated as a qualitative presence/absence endpoint for residual infectious virus; the microscopy images were not used as a quantitative assay.

### Buffer exchange did not affect virus titer

The inactivation reagents used in this study are known to be cytotoxic. Therefore, we performed a buffer exchange following sample inactivation to avoid cytotoxic effects that could affect viral replication. To evaluate the impact of the buffer exchange process on virus infectivity, infected cell samples prepared in Dulbecco's Phosphate-Buffered Saline (DPBS), with and without buffer exchange, were used (Fig 2), and plaque assays were performed to compare virus titers across samples. As shown in Fig 3, the column buffer exchange method showed no statistically significant change compared with the untreated stock virus (P = 0.1840).

**Fig 2:**
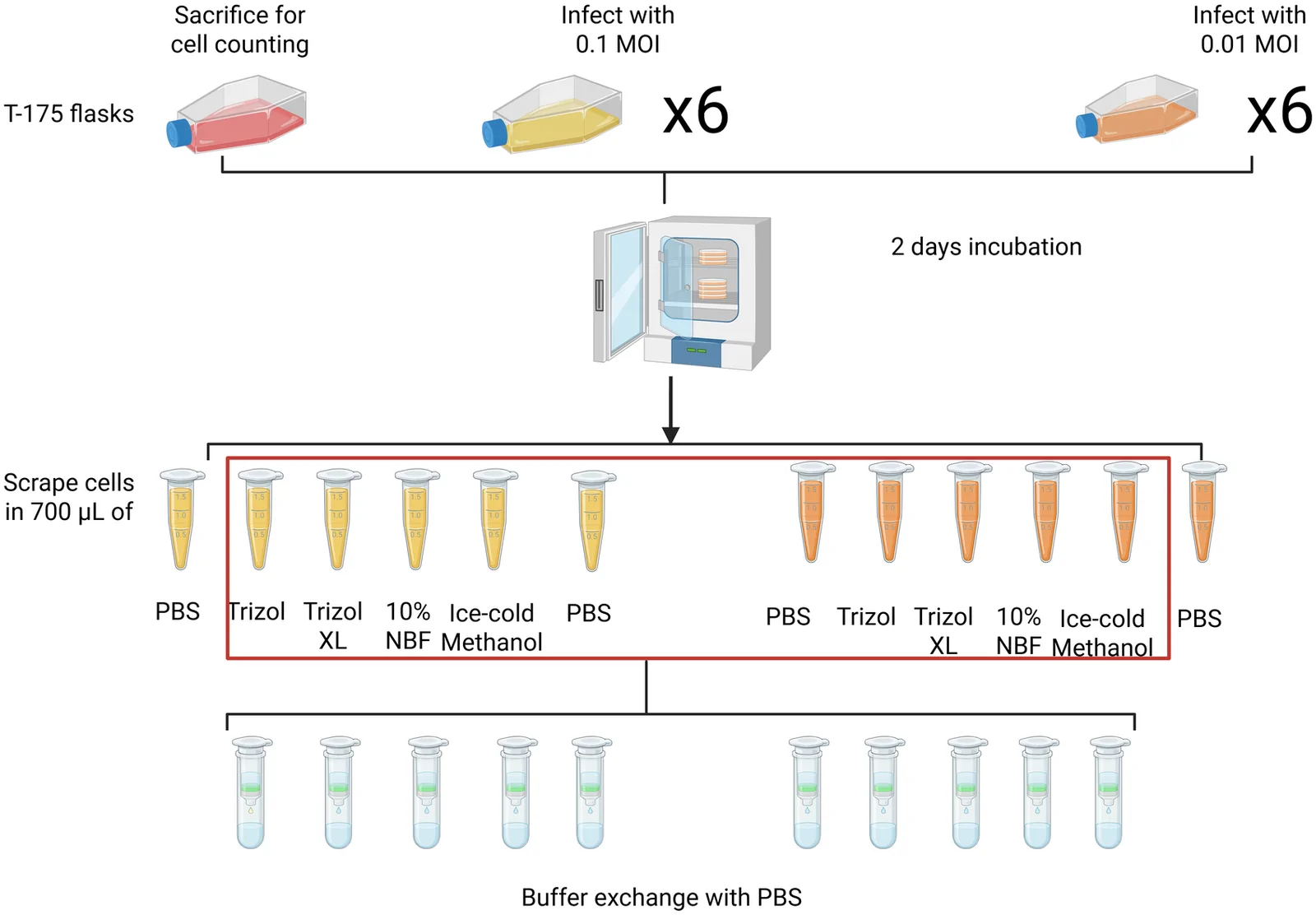
Schematic overview of chemical inactivation workflows for infected cell samples. Twelve T-175 flasks of confluent MDCK cells were infected with 0.1/0.01 MOI of H5N1 virus. After 2 dpi, the medium in the flasks was removed, and cells were collected with a cell scraper in 1 mL of 10% NBF, ice-cold methanol, TRIzol, or TRIzol LS following standard inactivation protocols. After the indicated inactivation steps, samples were transferred to permissive cell cultures to assess residual infectivity using cytopathic effect as the endpoint.

**Fig 3:**
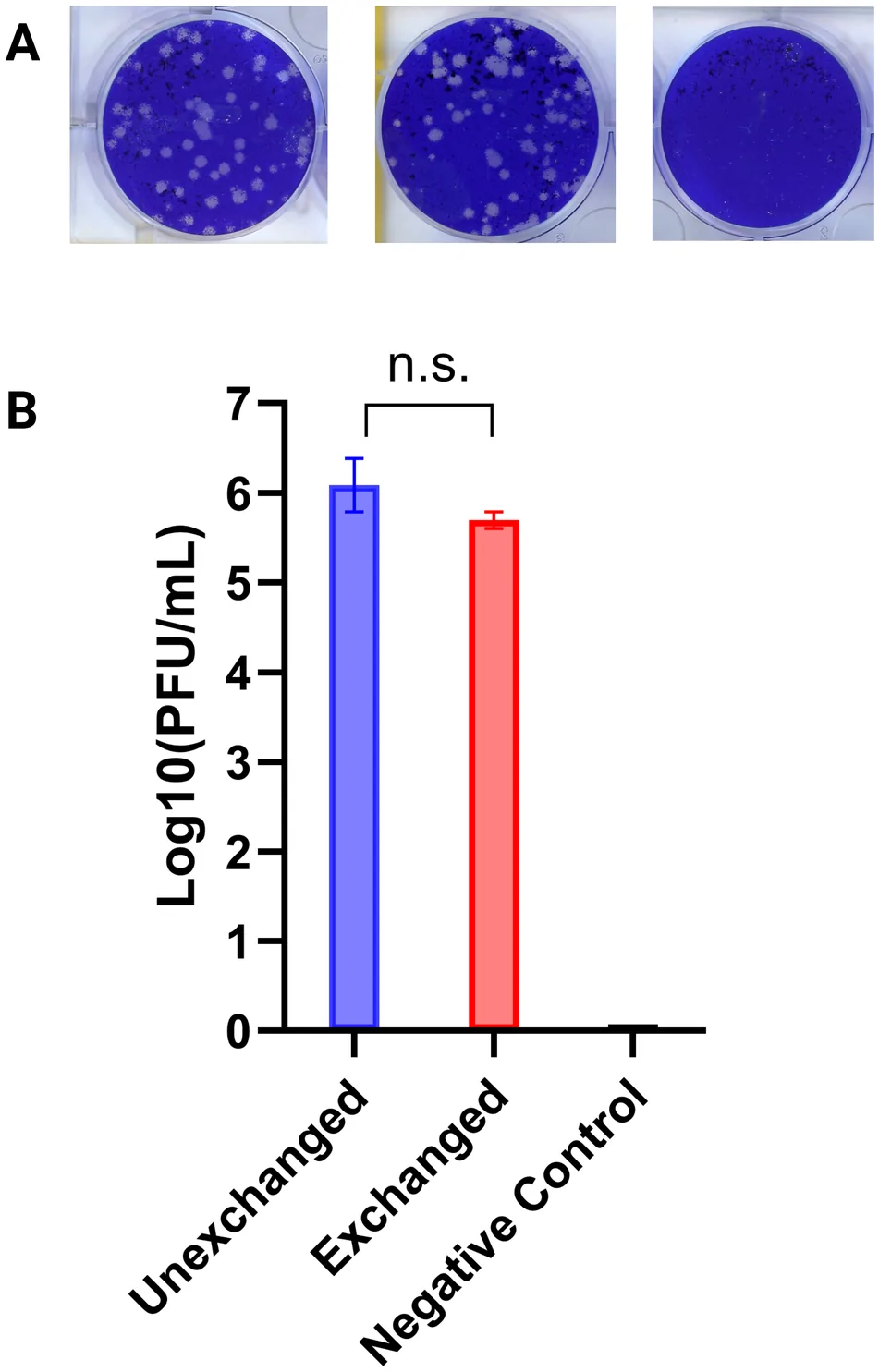
H5N1 virus titer before and after buffer exchange. A. Pictures of plaque assay for the non-buffer-exchanged sample (positive control), buffer-exchanged sample, and negative control at the same dilution factor. The non-buffer-exchanged infectious-virus sample served as the positive control/reference for assessing whether buffer exchange affected recovery of infectious virus. B. Statistical analysis of plaque assay was plotted by GraphPad Prism 11. The result was analyzed using Student’s t-test, with P = 0.1840.

### Treatment with the four inactivation reagents of different types of IAV-infected samples inactivated IAV infectious particles

To determine the effectiveness of the four inactivation reagents to inactivate IAV across different sample types, we first tested samples from infected cells, fluid, and homogenized tissue. Each sample was mixed with the four inactivation reagents. All experiments included positive and negative controls. As shown in Fig 4, no CPE was observed in inactivation reagent-treated samples at second blind passage. In contrast, CPE was observed for untreated positive controls, and all negative controls showed no CPE. All reagents (700 μL) inactivated up to 5.0 × 10^5^ PFU of H5N1 virus in 1.2 × 10^7^ MDCK cells (Table 1). TRIzol LS or ice-cold methanol with a volume of 750 μL inactivated up to 1.5 x 10^7^ PFU in 250 μL fluid samples and 5.0 x 10^7^ PFU in 250 μL homogenized tissue samples. For 910 μL of 10% NBF or TRIzol, inactivation was observed with up to 5.4 x 10^6^ PFU in 90 μL fluid samples and 1.8 x 10^7^ PFU in 90 μL homogenized tissue samples.

**Table 1:** Inactivation methods used in this study to inactivate samples containing IAV.

Sample	Inactivation chemical	Amount of IAV
IAV infected cells	10% NBF (700 μL) ice-cold methanol (700 μL) TRIzol (700 μL) TRIzol LS (700 μL)	5.0 x 10^5^ PFU (1.2 × 10^7^ cells)
IAV fluid	10% NBF (910 μL) ice-cold methanol (750 μL) TRIzol (910 μL) TRIzol LS (750 μL)	5.4 x 10^6^ PFU (90 μL) 1.5 x 10^7^ PFU (250 μL) 5.4 x 10^6^ PFU (90 μL) 1.5 x 10^7^ PFU (250 μL)
IAV infected mouse lung homogenate	10% NBF (910 μL) ice-cold methanol (750 μL) TRIzol (910 μL) TRIzol LS (750 μL)	1.8 x 10^7^ PFU (90 μL Tissue homogenate) 5.0 x 10^7^ PFU (250 μL Tissue homogenate) 1.8 x 10^7^ PFU (90 μL Tissue homogenate) 5.0 x 10^7^ PFU (250 μL Tissue homogenate)

**Fig 4:**
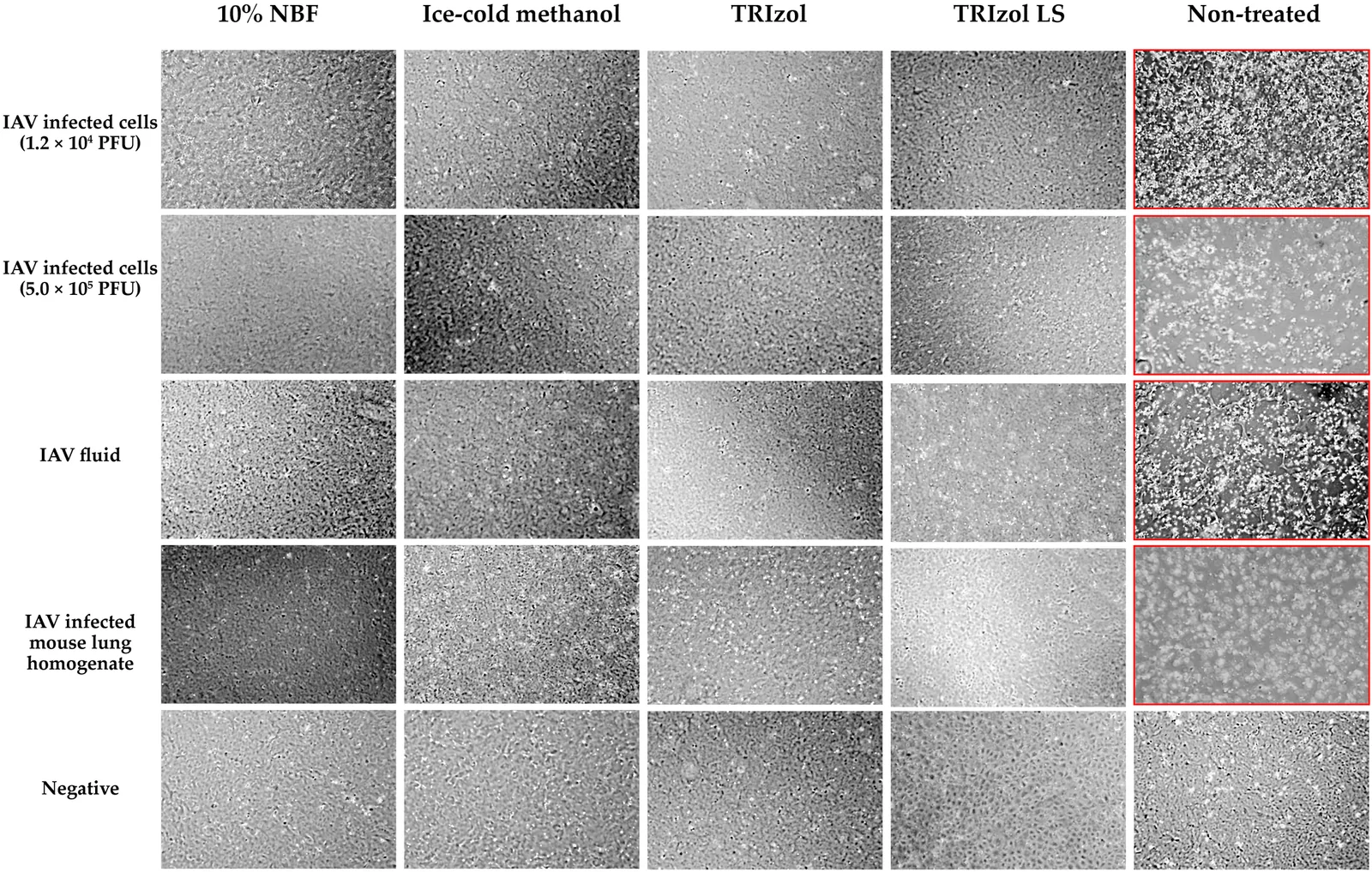
Representative microscope images (10x) of cells following the second blind passage with different inactivation treatments for different types of samples. Images showing CPE are outlined in red. CPE was evaluated as a qualitative presence/absence endpoint for residual infectious virus; therefore, the representative microscopy images were not subjected to image-based quantification or statistical analysis.

## Discussion

Laboratories generating Select Agent-containing samples for downstream analyses at lower biocontainment levels must have in-house-validated inactivation procedures [1,2]. For viruses, demonstrating the absence of detectable infectious virus can be challenging because the inactivation reagents are typically toxic to the indicator cells required to assess viral viability. Therefore, validation protocols must be carefully controlled to ensure that residual inactivation agent does not affect the sensitivity of the viability assay.

In this study, we first established the sensitivity of our approach using serial dilutions of H5N1 virus in MDCK cells. Two blind passages further increased the sensitivity of infectious virus detection, allowing detection as low as 0.1 PFU (Fig 1). An important component of this workflow was the incorporation of column-based buffer exchange before viability testing. The chemical inactivants tested are cytotoxic to MDCK cells, and if they remained in the infectivity assay, they could impact cell viability and reduce the sensitivity of virus detection. This creates a potentially serious risk of false negatives, in which residual infectious virus is undetected because the indicator cells are killed by residual inactivants. The buffer-exchange step was included to remove the inactivants before inoculation of MDCK cells. Crucially, we showed that this process did not significantly reduce the recovery of infectious virus. This indicated that the column step did not compromise assay sensitivity (Fig 3).

Using the viability testing protocol, treatment with 10% NBF, TRIzol, TRIzol LS, and ice-cold methanol rendered infectious virus undetectable from all the samples tested, including infected cell culture samples, virus-containing fluids, and infected tissues (Fig 4). Thus, the workflow combines chemical inactivation, removal of residual cytotoxic inactivants, and sensitive cell-based detection of infectivity. These findings are consistent with other studies showing that commonly used fixation and extraction reagents effectively inactivate enveloped viruses [5–10,15,16].

The primary endpoint of this study was the presence or absence of residual infectious virus. For this reason, cell-based infectivity testing by plaque assay and serial blind passage was prioritized over molecular or antigen-detection assays. RT-qPCR can detect viral RNA that remains after successful loss of infectivity, and immunostaining can similarly detect residual viral antigen without demonstrating replication-competent virus. Accordingly, these assays would provide complementary information on persistence of viral components but would not by themselves establish successful inactivation. Molecular or antigen-based assays may nevertheless be useful as orthogonal measurements in future studies.

For the infected lung tissue, PR8 (H1N1) was used as a surrogate influenza A virus rather than H5N1 HPAIV. The Federal Select Agent Program recognizes that viruses from the same family may be suitable surrogates during development of inactivation procedures when relevant inactivation properties are appropriately considered [1]. PR8 and H5N1 are both enveloped influenza A viruses in the family *Orthomyxoviridae* and share structural features relevant to the chemical treatments evaluated here. Nevertheless, the PR8 lung-tissue experiments should not be interpreted as direct validation of H5N1-infected lung tissue. Rather, they demonstrate applicability of the workflow to an influenza A virus-infected tissue and provide a framework for subsequent facility-specific validation with the intended HPAIV infected tissue, as appropriate.

In conclusion, these studies evaluated four chemical inactivation procedures using H5N1-containing cell culture and fluid samples and PR8-infected mouse lung homogenates. Our evaluation addresses an important need in biosafety workflows for influenza A viruses. PR8 was used as a surrogate influenza A virus to evaluate the inactivation and viability-testing workflow in tissue samples; therefore, the tissue experiments should not be interpreted as direct validation of H5N1-infected lung tissue. It is also important to note that inactivation validation is procedure- and facility-specific. The methodology described here can serve as a framework for designing and conducting facility-specific validation studies for HPAIV-containing samples, with validation using the intended virus and samples performed as appropriate.

## Materials and methods

### Ethics statement

This study was performed following standard operating procedures approved by the University of Missouri’s AAALAC-accredited animal biosafety level 3 (ABSL-3) laboratory at the Laboratory of Infectious Disease Research (LIDR). Experiments were performed following recommendations described in the Guide for the Care and Use of Laboratory Animals (National Research Council, 2011) and were approved by the University of Missouri Animal Care and Use Committee (IACUC) (IACUC protocol 63224; initial approval date: 23 January 2025; amendment approved: 4 March 2026; to RMO).

### Cells and viruses

Madin-Darby Canine Kidney cells (MDCK, NBL-2) (ATCC, Manassas, VA, USA) were maintained at 37 °C with 5% CO_2_ in growth media that consisted of Eagle's minimum essential medium (EMEM) (Gibco, Grand Island, NY, USA) containing 10% heat-inactivated fetal bovine serum (FBS) (Gibco, Grand Island, NY, USA). A/bovine/Ohio/B24OSU-439/2024 (H5N1 Clade 2.3.4.4b) and A/Puerto Rico/8/1934–Mouse Adapted (H1N1, PR8) viruses were acquired from BEI and amplified in our laboratory.

### Determination of the limit of detection (LOD)

MDCK cells maintained in EMEM supplemented with 10% FBS were seeded onto T-75 tissue culture flasks at a density of 2.5 × 10^6^ cells per flask and incubated at 37 °C and 5% CO_2_ overnight. To evaluate the sensitivity of the experimental design, we conducted an LOD assay (Fig 5). For infection, 0, 0.01, 0.1, 1, or 10 PFU of H5N1 virus were diluted in infection medium supplemented with 0.36% BSA and 15 µg TPCK-trypsin (Thermo Scientific, Manassas, VA, USA) for a final volume of 15 mL. These were used to inoculate the T-75 flask containing MDCK cells seeded the day prior. After 1 hour, the inoculum was removed, the cells were washed three times with 15 mL of DPBS, and fresh EMEM containing 0.36% BSA and 15 µg TPCK-trypsin was added to the cells. Cells were incubated for 2 days at 37 °C, and daily microscopy was performed to monitor CPE. At two dpi, the first blind passage was performed with all of the cell culture medium from each flask removed and used to inoculate fresh MDCK cells seeded the day prior in a 300 cm^2^ flask, along with fresh 35 mL EMEM, 0.36% BSA, and 50 µg TPCK-trypsin. After a further two days, a second blind passage was performed in the same manner, followed by daily monitoring for six days.

**Fig 5:**
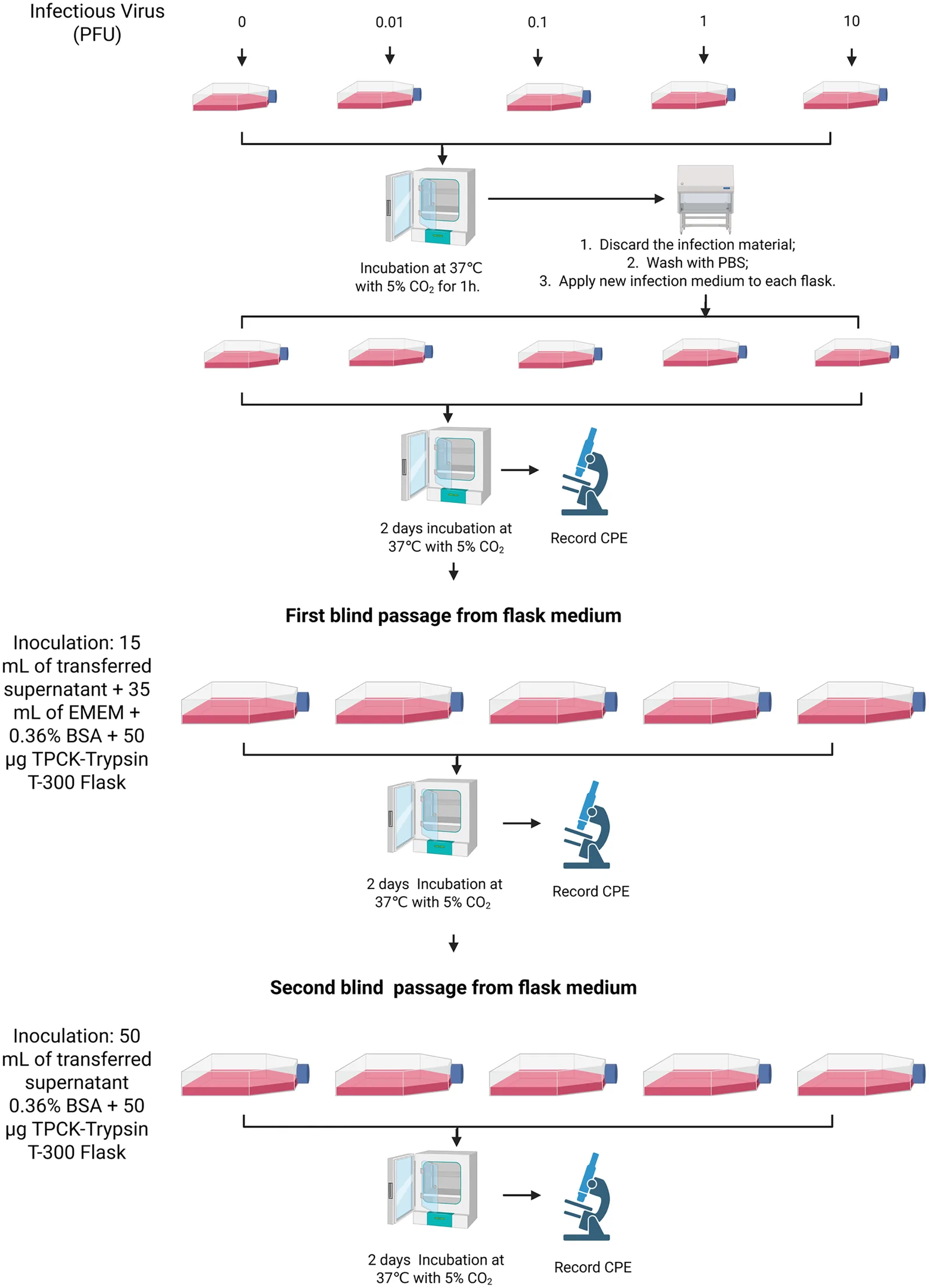
Experimental workflow to determine the limit of detection (LOD) of infectious IAV. Serial dilutions of H5N1 virus (target 0–10 PFU) were incubated on confluent cell monolayers for 1h, washed, and overlaid with EMEM containing 0.36% BSA and 15 µg TPCK-trypsin in T-75 flasks. Cultures were monitored for cytopathic effect for 2 days, followed by two rounds of blind passages using flask supernatants onto fresh cells, with an additional 2-day observation each round to assess infectious virus.

### Preparation of test samples

To generate infectious material for the inactivation validation studies, 12 T-175 flasks of confluent MDCK cells were infected with H5N1 virus. To ensure a high viral titer when the cells were harvested for inactivation, individual flasks were inoculated at multiplicities of infection (MOI) of either 0.1 or 0.01. At two days post-infection, the media in the flasks were removed. The cells were washed three times with DPBS, then 700 μL of inactivation chemical reagent or DPBS control was added. The monolayers were collected using a cell scraper (Fig 2). For fluid samples, we used H5N1 stock virus with a titer of 6.0 x 10^7^ PFU/mL.

To generate tissue samples, five 6-week-old female C57BL/6 mice were anesthetized with isoflurane prior to intranasal inoculation with 10⁴ PFU of PR8. Following infection, animals were monitored and weighed daily to assess their health and well-being and to minimize unnecessary suffering. A clinical score sheet was employed to monitor disease progression, and any animals that were determined to be moribund were immediately euthanized. At three days post-infection, mice were euthanized using CO_2_, and lungs were collected and immediately flash-frozen at −80 °C. For tissue homogenized in media, 100 mg of tissue was added to 1 mL of cell culture media and homogenized using a Qiagen TissueLyser II. Samples were clarified by low-speed centrifugation and transferred to new tubes [17]. Later, homogenized tissue samples were subjected to plaque assay, yielding a titer of 2.0 x 10^8^ PFU/mL.

### Virus recovery from specimens following buffer exchange

To verify the impact of buffer exchange on virus titer, we compared the virus titers with and without buffer exchange from the same sample preparation process (Fig 2). Pierce^®^ Zeba™ Spin Desalting Columns (2 mL) (Thermo Scientific, Manassas, VA, USA) were prepared according to the manufacturer’s instructions. Briefly, 700 µL of the sample was slowly applied to the center of the prepared column’s resin bed. After the sample had absorbed into the resin, 40 µL of DPBS was added to the resin. The column was centrifuged at 1000 × g for 2 min. The volume of the collected sample was brought to 700 µL for comparison. Plaque assays were performed on samples with and without buffer exchange to quantify the remaining infectious virus [11].

### Chemical inactivation of H5N1-infected cell samples

The inactivation agents used were 10% NBF (LabChem, Zelienople, PA, USA), TRIzol (Life Technologies, Carlsbad, CA, USA), 99% ice-cold methanol, and TRIzol LS (Life Technologies, Carlsbad, CA, USA) for infected cells, fluid, and homogenized tissue samples containing infectious IAV. For each reagent, a set of experimental samples, together with positive and negative controls were included.

For each infected cell sample treatment, 700 μL of an inactivation reagent was added to the 1.2 × 10^7^ infected cells in the flasks, which was then collected and transferred to 1.5 mL Eppendorf tubes (Fig 2). For the inactivation negative control, an Eppendorf tube was filled with 700 μL of DPBS. For the inactivation positive control, an Eppendorf tube was filled with 700 μL of DPBS containing 1.2 × 10^7^ infected cells. All the tubes were mixed vigorously and incubated for 10 min. Experimental cell samples were prepared by infecting cells at MOIs of 0.01 or 0.1 with H5N1 and then treating them for 10 min with the inactivation reagent. Cell samples infected with H5N1 at MOIs of 0.01 or 0.1 were used as positive controls. Non-infected cell culture samples were treated with the inactivation reagent for 10 min, and untreated cells served as negative controls. Then buffer exchange was performed for all samples.

### Chemical inactivation of H5N1 liquid samples

To inactivate IAV in fluid samples, 750 μL of ice-cold methanol or TRIzol LS reagent was added to 250 μL of 1.5 × 10^7^ PFU H5N1 virus in 1.5 mL Eppendorf tubes. For TRIzol or 10% NBF treatment, a volume ratio of 10 parts chemical to 1 part fluid sample was used (910 μL: 90 μL). For the inactivation negative control, an Eppendorf tube was filled with 1 mL of infection medium (EMEM + 0.36% BSA + 1 µg TPCK-trypsin). For the inactivation positive control, an Eppendorf tube was filled with 250 μL of 1.5 × 10^7^ PFU H5N1 and 750 μL of infection medium (EMEM + 0.36% BSA + 1 µg TPCK-trypsin). All the tubes were mixed vigorously and incubated for 10 minutes. The liquid sample containing 1.5 × 10^7^ PFU H5N1 was used as a positive control. Non-infected cell culture medium samples treated for 10 min with an inactivation reagent and untreated medium were used as negative controls. Then buffer exchange was performed for all samples.

### Chemical inactivation of PR8-infected mouse lung tissue samples

For lung tissue samples, 750 μL of ice-cold methanol or TRIzol LS was mixed with 250 μL of homogenized tissue in 1.5 mL Eppendorf tubes. For TRIzol or 10% NBF treatment, a volume ratio of 10 parts chemical to 1 part fluid sample was used (910 μL: 90 μL). For the inactivation negative control, one Eppendorf tube was filled with 1 mL of infection medium (EMEM + 0.36% BSA + 1 µg TPCK-trypsin). For the inactivation positive control, an Eppendorf tube was filled with 250 μL of centrifuged sample and 750 μL of infection medium (EMEM + 0.36% BSA + 1 µg TPCK-trypsin). The mixture was vortexed and incubated at room temperature for 10 min. Then buffer exchange was performed for all samples.

### Determination of virus titer

MDCK cells maintained in EMEM supplemented with 10% FBS were seeded into 6-well plates at a density of 8.0 × 10^5^ cells per well and incubated at 37 °C and 5% CO_2_ overnight. Tenfold serial dilutions, from no dilution to 10^-5^ of the virus stock or test samples, were prepared in infection media. Growth medium was removed, the cells were washed once with DPBS, and then 200 µL of each virus dilution was added. Plates were incubated at 37 °C and 5% CO_2_ for 1 h. Cells were then overlaid with 2 mL of a 1:1 solution of 2.5% Avicel RC-591 and 2 × Temin's Modified Eagle Medium without phenol red, supplemented with 0.36% BSA and 1 µg/mL TPCK-trypsin, and incubated at 37 °C and 5% CO_2_. After 3 days, plates were fixed with 10% NBF and stained with 0.2% Gentian Violet in 10% NBF. The number of plaques per virus dilution was used to enumerate infectious virus [15].

### Analysis of samples after inactivation using serial blind passages

MDCK cells maintained in EMEM supplemented 10% FBS were seeded into T-75 tissue culture flasks at a density of 2.5 × 10^6^ cells per flask and incubated at 37 °C and 5% CO_2_ overnight. The chemically inactivated samples described above were used to inoculate the flasks (Fig 6). Briefly, 1 mL of each sample was diluted in 14 mL of infection medium. The viral inoculum was added to the cells, and after one hour of incubation, it was removed. The cells were washed thrice with 15 mL of DPBS, and fresh infection medium was added. Cells were incubated for 2 days at 37 °C and were monitored for CPE. Then, all cell culture media from each flask were collected and used to inoculate fresh MDCK cells seeded the day prior in a T-300 flask, with the entire volume from one flask deposited into a single flask along with 35 mL of infection medium, 0.36% BSA, and 50 µg TPCK-trypsin. Observation and imaging of these cells continued until day 2 post-inoculation. The second blind passage was performed identically to the first.

**Fig 6:**
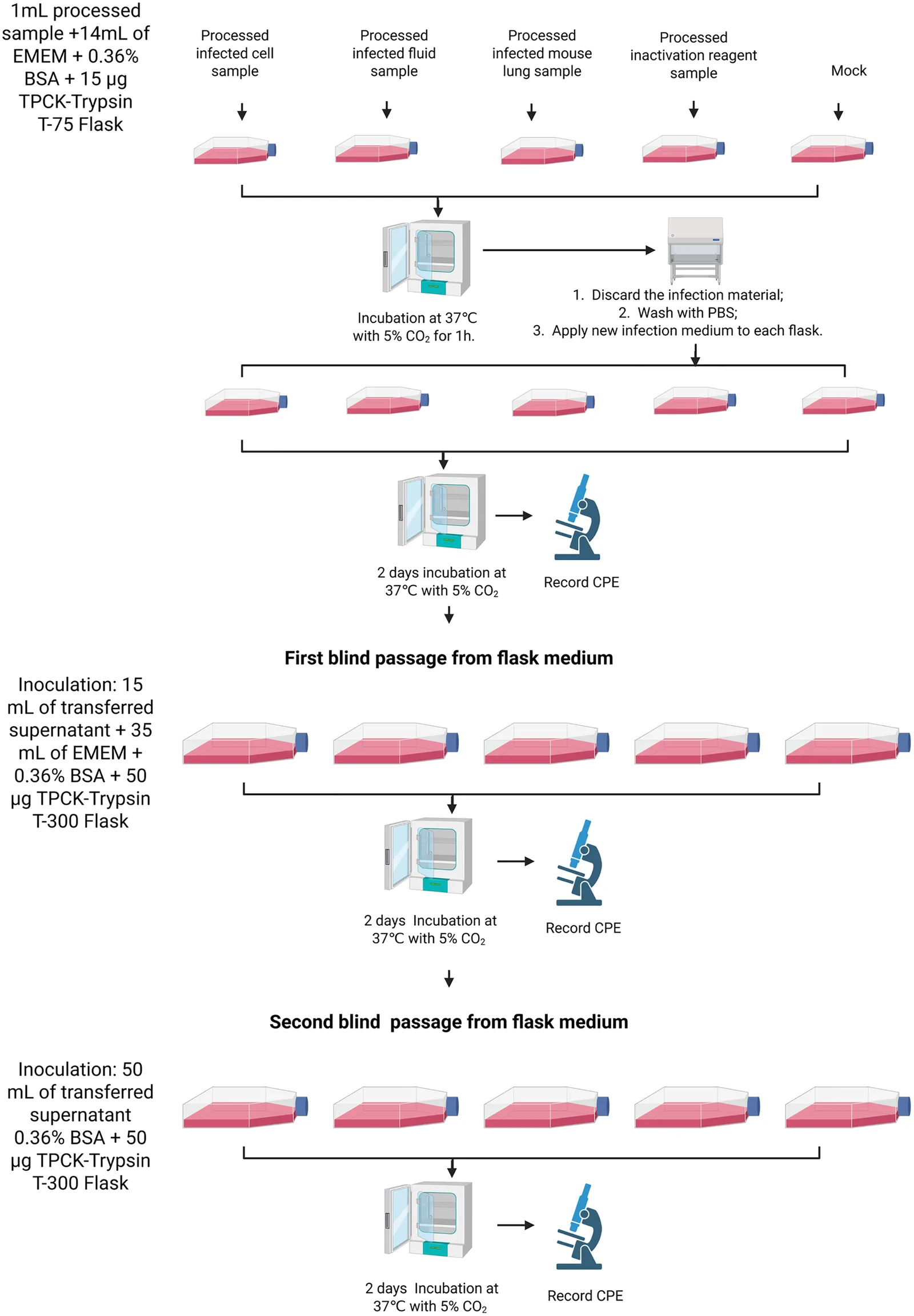
Schematic overview of chemical inactivation workflows for different types of samples. Test IAV-infected cells, IAV fluid, or IAV-infected mouse lung tissue were treated with 10% NBF, ice-cold methanol, TRIzol, or TRIzol LS following standard inactivation protocols. After the indicated inactivation pipelines, samples were transferred to permissive cell cultures to assess residual infectivity by CPE.

### Data analysis

Plaque assay data were analyzed and plotted using GraphPad Prism 11. Differences between buffer-exchanged and non-buffer-exchanged samples were analyzed using Student’s t-test. A P value ≤ 0.05 was considered statistically significant.
