# Supplementary material for: Validation of chemical inactivation methods for Select Agent compliance using HPAIV and low-pathogenicity influenza A virus in tissues and cultured cells
Source: PLoS One. 2026 Nov 6;21(11):e0362117. doi: 10.1371/journal.pone.0362117 (PMC13742933; doi:10.1371/journal.pone.0362117)
Supplement: Peer review history

# Peer review: Decision Letter 0

**DOI:** 10.1371/journal.pone.0362117.r001

**Authors:** Shih-Chao Lin

29 Sep 2026

Dear Dr. Griffiths,

Thank you for submitting your manuscript to PLOS One. After careful consideration, we feel that it has merit but does not fully meet PLOS One’s publication criteria as it currently stands. Therefore, we invite you to submit a revised version of the manuscript that addresses the points raised during the review process.

Please submit your revised manuscript by Oct 29 2026 11:59PM. If you will need more time than this to complete your revisions, please reply to this message or contact the journal office at plosone@plos.org. When you're ready to submit your revision, log on to https://www.editorialmanager.com/pone/ and select the 'Submissions Needing Revision' folder to locate your manuscript file.

- A letter that responds to each point raised by the academic editor and reviewer(s). You should upload this letter as a separate file labeled 'Response to Reviewers'.
- A marked-up copy of your manuscript that highlights changes made to the original version. You should upload this as a separate file labeled 'Revised Manuscript with Track Changes'.
- An unmarked version of your revised paper without tracked changes. You should upload this as a separate file labeled 'Manuscript'.

If you would like to make changes to your financial disclosure, please include your updated statement in your cover letter. Guidelines for resubmitting your figure files are available below the reviewer comments at the end of this letter.

If applicable, we recommend that you deposit your laboratory protocols in protocols.io to enhance the reproducibility of your results. Protocols.io assigns your protocol its own identifier (DOI) so that it can be cited independently in the future. For instructions see: https://journals.plos.org/plosone/s/submission-guidelines#loc-laboratory-protocols. Additionally, PLOS One offers an option for publishing peer-reviewed Lab Protocol articles, which describe protocols hosted on protocols.io. Read more information on sharing protocols at https://plos.org/protocols?utm_medium=editorial-email&utm_source=authorletters&utm_campaign=protocols.

As the corresponding author, your ORCID iD is verified in the submission system and will appear in the published article. PLOS supports the use of ORCID, and we encourage all coauthors to register for an ORCID iD and use it as well. Please encourage your coauthors to verify their ORCID iD within the submission system before final acceptance, as unverified ORCID iDs will not appear in the published article. *Only*  the individual author can complete the verification step; PLOS staff *cannot*  verify ORCID iDs on behalf of authors.

We look forward to receiving your revised manuscript.

Kind regards,

Shih-Chao Lin, Ph.D.

Academic Editor

PLOS One

Journal Requirements:

When submitting your revision, we need you to address these additional requirements.

1. Please ensure that your manuscript meets PLOS ONE's style requirements, including those for file naming. The PLOS ONE style templates can be found at

https://journals.plos.org/plosone/s/file?id=wjVg/PLOSOne_formatting_sample_main_body.pdf and

https://journals.plos.org/plosone/s/file?id=ba62/PLOSOne_formatting_sample_title_authors_affiliations.pdf

2. To comply with PLOS One submissions requirements, in your Methods section, please provide additional information regarding the experiments involving animals and ensure you have included details on (1) methods of sacrifice, (2) methods of anesthesia and/or analgesia, and (3) efforts to alleviate suffering.

3. Please update your submission to use the PLOS LaTeX template. The template and more information on our requirements for LaTeX submissions can be found at http://journals.plos.org/plosone/s/latex.

4. We note that the grant information you provided in the ‘Funding Information’ and ‘Financial Disclosure’ sections do not match.

When you resubmit, please ensure that you provide the correct grant numbers for the awards you received for your study in the ‘Funding Information’ section.

5. Thank you for stating the following financial disclosure:

“UC7AI180306 (Griffiths, PI)”

Please state what role the funders took in the study.  If the funders had no role, please state: "The funders had no role in study design, data collection and analysis, decision to publish, or preparation of the manuscript."

If this statement is not correct you must amend it as needed.

Please include this amended Role of Funder statement in your cover letter; we will change the online submission form on your behalf.

6. If the reviewer comments include a recommendation to cite specific previously published works, please review and evaluate these publications to determine whether they are relevant and should be cited. There is no requirement to cite these works unless the editor has indicated otherwise.

7. Please review your reference list to ensure that it is complete and correct. If you have cited papers that have been retracted, please include the rationale for doing so in the manuscript text, or remove these references and replace them with relevant current references. Any changes to the reference list should be mentioned in the rebuttal letter that accompanies your revised manuscript. If you need to cite a retracted article, indicate the article’s retracted status in the References list and also include a citation and full reference for the retraction notice.

[Note: HTML markup is below. Please do not edit.]

Reviewers' comments:

Reviewer's Responses to Questions

**Comments to the Author**

1. Is the manuscript technically sound, and do the data support the conclusions?

Reviewer #1: Yes

Reviewer #2: Partly

Reviewer #3: Partly

**********

2. Has the statistical analysis been performed appropriately and rigorously?

Reviewer #1: Yes

Reviewer #2: No

Reviewer #3: Yes

**********

3. Have the authors made all data underlying the findings in their manuscript fully available?

The PLOS Data policy

Reviewer #1: Yes

Reviewer #2: Yes

Reviewer #3: Yes

**********

4. Is the manuscript presented in an intelligible fashion and written in standard English?

Reviewer #1: Yes

Reviewer #2: Yes

Reviewer #3: Yes

**********

Reviewer #1: I appreciate the protocol design by the authors in the manuscript. The manuscript is beneficial for the readers in the virology filed particularly working with select agents. However, I suggest the authors to cite any previous study involving protocol design for inactivating IAV if available. Also, how the present study is unique from the other studies reported.

Reviewer #2: Comments on “ Validation of Chemical Inactivation Methods for Select Agent Compliance Using HPAIV and Low-Pathogenicity Influenza A Virus in Tissues and Cultured Cells”

The manuscript is well- written. It presents the findings of the study in an intelligible and scientific manner. There are few minor issues that I would like to mention to be addressed in order to be accepted for publication. The following are the ones.

1. Fig 1,4 - Any quantification of the images attached, if possible to be added and statistical validation to be accompanied.

2. Fig 3 – The labelling of the wells presented needs to be clearer. The graph has a negative control, but it would be better if a positive control could also be added.

Reviewer #3: The article has merit, as it validates inactivation methods for the influenza virus. The methodology is adequate; however, some clarifications are needed.

Although virus isolation is the gold-standard test for viral diagnosis, why was no other test performed, such as real-time PCR or an immunostaining assay, to identify the agent after the inactivation tests? This would add robustness to the study.

Why was the high-pathogenicity sample not used in lung tissue? Under the conditions presented, the conclusion that "The methodology and procedures described here can serve as a framework for designing and conducting facility-specific validation studies for HPAIV workflows" applies only to infected cells and fluids, whereas for lung tissue only the PR8 sample was used.

One point that should be clarified: in the Materials and Methods, it is stated that the study was approved by IACUC protocol 63224, approval date: 04 March 2026, to RMO. Were the mice used for inoculation and tissue collection handled after this date?

Why were the samples incubated with the inactivating agents for only 10 minutes? Why was a time gradient not performed? Is there any previous study demonstrating that 10 minutes is the optimal time? If so, please cite it.

**********

what does this mean?). If published, this will include your full peer review and any attached files.

If you choose “no”, your identity will remain anonymous but your review may still be made public.

**Do you want your identity to be public for this peer review?**  For information about this choice, including consent withdrawal, please see our Privacy Policy

Reviewer #1: No

Reviewer #2: No

Reviewer #3: No

**********

[NOTE: If reviewer comments were submitted as an attachment file, they will be attached to this email and accessible via the submission site. Please log into your account, locate the manuscript record, and check for the action link "View Attachments". If this link does not appear, there are no attachment files.]

To ensure your figures meet our technical requirements, please review our figure guidelines: https://journals.plos.org/plosone/s/figures

You may also use PLOS’s free figure tool, NAAS, to help you prepare publication quality figures: https://journals.plos.org/plosone/s/figures#loc-tools-for-figure-preparation.

NAAS will assess whether your figures meet our technical requirements by comparing each figure against our figure specifications.

# Author comment: Author response to Decision Letter 1

**DOI:** 10.1371/journal.pone.0362117.r002

13 Oct 2026

Reviewer #1:

I appreciate the protocol design by the authors in the manuscript. The manuscript is beneficial for the readers in the virology field, particularly working with select agents. However, I suggest the authors to cite any previous study involving protocol design for inactivating IAV if available. Also, how the present study is unique from the other studies reported.

Response:

We sincerely thank the reviewer for the positive assessment of our manuscript and for this important suggestion. We agree that additional discussion of previously published IAV inactivation studies would help place our work in the context of the existing literature.

Several previous studies have evaluated the inactivation of influenza A virus using chemical or sample-processing reagents. For example, Weber and Stilianakis reviewed methods for influenza A virus inactivation and environmental persistence [5]. Jeong et al. evaluated the inactivation of H1N1 influenza A virus using disinfectants [7], while Avelin et al. evaluated the inactivation of H5N1 avian influenza virus using commonly used sample-preparation reagents, including methanol, paraformaldehyde, and RNA extraction reagents [8]. Abad also evaluated inactivation procedures using H7N1 HPAIV suspensions [9], and Kochel et al. investigated TRIzol LS-mediated inactivation of multiple viruses [10]. These studies have now been more explicitly discussed in the revised manuscript.

The present study differs from these previous investigations in several important respects. First, our primary objective was not simply to demonstrate that a chemical reagent can inactivate IAV, but to establish a facility-specific validation workflow appropriate for samples generated during Select Agent/HPAIV research. As emphasized in the manuscript, inactivation procedures must be validated under the specific conditions in which they are used because residual chemical reagents can interfere with cell-based infectivity assays and potentially produce false-negative results.

Second, our study evaluated multiple types of biologically relevant samples, including H5N1-infected cell cultures, virus-containing fluids, and PR8-infected mouse lung homogenates, rather than evaluating a single virus preparation. The study therefore addresses the influence of sample types and provides a framework that can be adapted to different laboratory workflows.

Third, an important component of our validation strategy was the use of column-based buffer exchange following chemical treatment to remove residual cytotoxic inactivation reagents before the viability assay. We experimentally demonstrated that this buffer-exchange step did not significantly reduce recovery of infectious virus (P = 0.1840), thereby supporting the sensitivity of the downstream viability assay.

Finally, we established the analytical sensitivity of the viability-testing workflow using a limit-of-detection experiment and demonstrated detection of infectious H5N1 at levels as low as 0.1 PFU following serial blind passage. Thus, the novelty of the present study lies primarily in the integration of sensitivity determination, chemical inactivation, removal of cytotoxic reagents, positive/negative controls, and serial blind passage into a facility-specific validation framework applicable to multiple IAV sample types.

We have revised the Introduction and Discussion to better acknowledge previous IAV inactivation studies and to clarify the specific contribution and novelty of the present work (line 59 -66; line 157-183).

Reviewer #2:

Comment:

The manuscript is well-written. It presents the findings of the study in an intelligible and scientific manner. There are few minor issues that I would like to mention to be addressed in order to be accepted for publication.

Response:

We thank the reviewer for the positive assessment of our manuscript and for the constructive comments. We have carefully considered the reviewer’s suggestions and revised the manuscript and figures accordingly, as detailed below.

Comment 1:

Fig. 1, 4 – Any quantification of the images attached, if possible to be added and statistical validation to be accompanied.

Response:

We thank the reviewer for this suggestion. Figures 1 and 4 are intended to provide representative endpoint images documenting the presence or absence of cytopathic effect (CPE), rather than to serve as quantitative imaging assays. The primary endpoint for these experiments was detection of residual infectious virus, assessed by CPE following serial blind passage.

For Fig. 1, CPE was observed following the first blind passage for samples inoculated with 1 and 10 PFU and following the second blind passage for the sample inoculated with 0.1 PFU, whereas no CPE was observed for the 0.01-PFU or uninfected controls. For Fig. 4, no CPE was observed in chemically treated samples, whereas CPE was observed in untreated positive controls and not in negative controls.

Because these experiments were designed as qualitative infectivity endpoints rather than quantitative image-analysis assays, image-based quantification and statistical analysis were not performed. We have clarified this in the revised figure legends.

Comment 2:

Fig 3 – The labelling of the wells presented needs to be clearer. The graph has a negative control, but it would be better if a positive control could also be added.

Response:

We thank the reviewer for this helpful comment. We apologize that the labeling in the original figure was not sufficiently clear. The “unexchanged” sample shown in Fig. 3 serves as the positive control in this experiment. This sample contained infectious H5N1 virus and was processed in parallel with the buffer-exchanged sample but was not subjected to the buffer-exchange procedure. It therefore provides the reference for determining whether the buffer-exchange step affects recovery of infectious virus. The negative control consisted of uninfected material and was included to confirm the absence of nonspecific plaque formation. We have revised the figure legend to explicitly identify the unexchanged sample as the positive control and to make the experimental groups clearer (line104-109).

Reviewer #3:

Comment 1:

The article has merit, as it validates inactivation methods for the influenza virus. The methodology is adequate; however, some clarifications are needed.

1. Although virus isolation is the gold-standard test for viral diagnosis, why was no other test performed, such as real-time PCR or an immunostaining assay, to identify the agent after the inactivation tests? This would add robustness to the study.

Response:

We thank the reviewer for this important suggestion. We agree that molecular and antigen-based assays can provide useful complementary information regarding the persistence of viral RNA or viral antigen following chemical treatment. However, the primary objective of this study was to validate chemical inactivation with respect to loss of infectious virus. RT-qPCR can detect viral RNA that remains intact or partially intact after successful inactivation, and immunostaining may detect residual viral antigen without demonstrating replication-competent virus. Therefore, these assays would not by themselves establish successful inactivation. For this reason, we prioritized cell-based infectivity testing and serial blind passage as the primary endpoint. We have clarified this rationale in the revised Discussion. (line 157-164).

Comment 2:

2. Why was the high-pathogenicity sample not used in lung tissue? Under the conditions presented, the conclusion that "The methodology and procedures described here can serve as a framework for designing and conducting facility-specific validation studies for HPAIV workflows" applies only to infected cells and fluids, whereas for lung tissue only the PR8 sample was used.

Response:

We thank the reviewer for this important comment and agree that the distinction between the H5N1 and PR8 experiments should be stated clearly. In the present study, H5N1 HPAIV was used to evaluate inactivation of infected cell culture material and virus-containing fluid samples, whereas A/Puerto Rico/8/1934 (H1N1; PR8) was used for the infected mouse lung homogenate experiments.

We used PR8-infected lung homogenate to evaluate the performance of the inactivation and viability-testing workflow in an animal-derived influenza A virus tissue matrix. PR8 and H5N1 are both enveloped influenza A viruses in the family Orthomyxoviridae and therefore share structural features relevant to the chemical inactivation approaches evaluated here. In addition, Federal Select Agent Program guidance recognizes that surrogate organisms, including viruses from the same family, may be useful during development of inactivation procedures when relevant inactivation properties are appropriately considered.

We agree, however, that the PR8 lung-tissue experiments do not constitute direct experimental validation of H5N1-infected lung tissue. We have therefore revised the manuscript to make clear that H5N1 was directly evaluated in infected cell culture and liquid virus samples, whereas PR8 was used as a surrogate influenza A virus tissue matrix. The revised text states that the PR8 tissue experiments demonstrate applicability of the workflow to an influenza A virus-infected tissue sample and provide a framework for subsequent facility-specific validation using the intended HPAIV-infected tissue when required. We have revised the manuscript to make this distinction explicit and to avoid implying that H5N1-infected lung tissue was directly evaluated in the present study (line 165-183).

Comment 3:

3. One point that should be clarified: in the Materials and Methods, it is stated that the study was approved by IACUC protocol 63224, approval date: 04 March 2026, to RMO. Were the mice used for inoculation and tissue collection handled after this date?

Response:

We thank the reviewer for identifying this point and apologize for the ambiguity in the original manuscript. IACUC protocol 63224 was initially approved on January 23, 2025. The date of March 4, 2026 reported in the original manuscript corresponds to the approval date of a subsequent amendment to this protocol, rather than the date of initial protocol approval. The animal experiments described in this study were conducted under the approved protocol. We have revised the Ethics Statement in the Materials and Methods section to clearly distinguish the initial protocol approval date from the amendment approval date (line 191-193).

Comment 4:

Why were the samples incubated with the inactivating agents for only 10 minutes? Why was a time gradient not performed? Is there any previous study demonstrating that 10 minutes is the optimal time? If so, please cite it.

Response:

The 10-minute incubation period was selected based on prior inactivation-validation work from our group using related chemical inactivation workflows, including Silva-Ayala and Griffiths, 2026. The objective of the present study was not to determine the minimum exposure time required for inactivation, but to validate whether the selected exposure condition eliminated detectable infectious virus under the specific sample-processing conditions used in our facility. We agree that a systematic time-course experiment would be valuable for determining the minimum effective exposure time, and we have clarified this distinction in the revised manuscript. (line 180-183).

Attachment Submitted filename: Response to Reviewers.docx

# Editor report: Decision Letter 1

**DOI:** 10.1371/journal.pone.0362117.r003

**Authors:** Shih-Chao Lin

20 Oct 2026

Validation of Chemical Inactivation Methods for Select Agent Compliance Using HPAIV and Low-Pathogenicity Influenza A Virus in Tissues and Cultured Cells

PONE-D-26-42742R1

Dear Dr. Griffiths,

We’re pleased to inform you that your manuscript has been judged scientifically suitable for publication and will be formally accepted for publication once it meets all outstanding technical requirements.

Within one week, you’ll receive an e-mail detailing the required amendments. When these have been addressed, you’ll receive a formal acceptance letter and your manuscript will be scheduled for publication.

An invoice will be generated when your article is formally accepted. Please note, if your institution has a publishing partnership with PLOS and your article meets the relevant criteria, all or part of your publication costs will be covered. Please make sure your user information is up-to-date by logging into Editorial Manager at Editorial Manager® and clicking the ‘Update My Information' link at the top of the page. For questions related to billing, please contact billing support.

If your institution or institutions have a press office, please notify them about your upcoming paper to help maximize its impact. If they’ll be preparing press materials, please inform our press team as soon as possible -- no later than 48 hours after receiving the formal acceptance. Your manuscript will remain under strict press embargo until 2 pm Eastern Time on the date of publication. For more information, please contact onepress@plos.org.

Kind regards,

Shih-Chao Lin, Ph.D.

Academic Editor

PLOS One

Additional Editor Comments (optional):

Reviewers' comments:

# Editor report: Acceptance letter

**DOI:** 10.1371/journal.pone.0362117.r004

**Authors:** Shih-Chao Lin

PONE-D-26-42742R1

PLOS One

Dear Dr. Griffiths,

I'm pleased to inform you that your manuscript has been deemed suitable for publication in PLOS One. Congratulations! Your manuscript is now being handed over to our production team.

At this stage, our production department will prepare your paper for publication. This includes ensuring the following:

* All references, tables, and figures are properly cited

* All relevant supporting information is included in the manuscript submission,

* There are no issues that prevent the paper from being properly typeset

You will receive further instructions from the production team, including instructions on how to review your proof when it is ready. Please keep in mind that we are working through a large volume of accepted articles, so please give us a few days to review your paper and let you know the next and final steps.

Lastly, if your institution or institutions have a press office, please let them know about your upcoming paper now to help maximize its impact. If they'll be preparing press materials, please inform our press team within the next 48 hours. Your manuscript will remain under strict press embargo until 2 pm Eastern Time on the date of publication. For more information, please contact onepress@plos.org.

You will receive an invoice from PLOS for your publication fee after your manuscript has reached the completed accept phase. If you receive an email requesting payment before acceptance or for any other service, this may be a phishing scheme. Learn how to identify phishing emails and protect your accounts at https://explore.plos.org/phishing.

If we can help with anything else, please email us at customercare@plos.org.

Thank you for submitting your work to PLOS One and supporting open access.

Kind regards,

PLOS One Editorial Office Staff

on behalf of

Dr. Shih-Chao Lin

Academic Editor

PLOS One
